# CTRP2 Overexpression Improves Insulin and Lipid Tolerance in Diet-Induced Obese Mice

**DOI:** 10.1371/journal.pone.0088535

**Published:** 2014-02-20

**Authors:** Jonathan M. Peterson, Marcus M. Seldin, Stefanie Y. Tan, G. William Wong

**Affiliations:** 1 Department of Physiology, The Johns Hopkins University School of Medicine, Baltimore, Maryland, United States of America; 2 Center for Metabolism and Obesity Research, The Johns Hopkins University School of Medicine, Baltimore, Maryland, United States of America; Consiglio Nazionale delle Ricerche, Italy

## Abstract

CTRP2 is a secreted plasma protein of the C1q family that enhances glycogen deposition and fat oxidation in cultured myotubes. Its *in vivo* metabolic function, however, has not been established. We show here that acute and chronic metabolic perturbations induced by fasting or high-fat feeding up-regulated the mRNA expression of *Ctrp2* in white adipose tissue without affecting its circulating plasma levels. We generated a transgenic mouse model with elevated circulating levels of CTRP2 to determine its metabolic function *in vivo*. When fed a low-fat diet, wild-type and CTRP2 transgenic mice exhibited no metabolic phenotypes. When challenged with a high-fat diet to induce obesity, wild-type and CTRP2 transgenic mice had similar weight gain, adiposity, food intake, metabolic rate, and energy expenditure. Fasting serum lipid and adipokine profiles were also similar between the two groups of mice. However, while glucose and insulin levels in the fasted state were comparable between wild-type and CTRP2 transgenic mice, insulin levels in the fed state were consistently lower in transgenic mice. Notably, CTRP2 transgenic mice had improved insulin tolerance and a greater capacity to handle acute lipid challenge relative to littermate controls. Our results highlight, for the first time, the *in vivo* role of CTRP2 in modulating whole-body metabolism.

## Introduction

C1q/TNF-related proteins (CTRPs) are secreted proteins with notable metabolic functions [1], [2], [3], [4], [5], [6], [7], [8]. CTRPs, and the insulin-sensitizing adipokine adiponectin, belong to the C1q family, resemble each other in overall domain structure and organization, and share sequence homology with the globular domain of immune complement C1q [9]. Each CTRP has a unique tissue expression profile and most circulate in plasma as multimeric glycoproteins [9]. Functional studies of CTRPs in mice suggest non-redundant metabolic, vasculoprotective, and cardioprotective functions for this class of secreted hormones [4], [7], [10], [11], [12], [13], [14], [15], [16], [17], [18], [19].

We identified CTRP2 as a secreted protein homologous to adiponectin [1]. CTRP2 shares 42% amino acid identity with adiponectin at the presumed functional globular C1q domain [1] and is expressed predominantly in adipose tissue. It also circulates as a trimeric glycoprotein in plasma [3]. Expression of *Ctrp2* transcript is up-regulated in young (8-week-old) but not older (12-week-old) leptin-deficient *ob/ob* mice; this is thought to be a compensatory response to leptin deficiency prior to the development of morbid obesity and severe insulin resistance [3]. Recombinant CTRP2 activates the conserved energy sensor AMP-activated protein kinase (AMPK) in a dose-dependent manner, similar to adiponectin [1]. Both the full-length protein and the truncated globular form of CTRP2 enhance fatty acid oxidation, as well as glycogen deposition, in differentiated mouse C2C12 myotubes [1]. Our previous *in vitro* study suggests a role for CTRP2 in regulating carbohydrate and lipid metabolism; its *in vivo* metabolic function, however, has not been established. In contrast, several related CTRPs have recently been shown to play important roles in controlling glucose and/or lipid metabolism in mice [4], [7], [10], [11], [12], [13]. In the current study, we used a transgenic mouse model to examine the *in vivo* metabolic function of CTRP2 in regulating energy balance.

## Materials and Methods

### Antibodies and Chemicals

Rat monoclonal anti-HA antibody (clone 3F10) was obtained from Roche Applied Science. Rabbit polyclonal anti-CTRP2 antibody was obtained from ProSci Inc. (Poway, CA; catalog no. 3561). Beta-tubulin HRP-conjugated antibody was obtained from Abcam (Cambridge, MA; catalog no. ab21058). Rabbit polyclonal antibodies recognizing phospho-AMPK (Thr-172), AMPK, phospho-ACC (Ser-79), and ACC were obtained from Cell Signaling Technology (Danvers, MA).

### Animals

C57BL/6J male mice (The Jackson Laboratory, Bar Harbor, ME) were used to evaluate diet-induced changes in *Ctrp2* mRNA and circulating levels. Sera were obtained from wild-type male mice following *ad libitum* feeding or overnight (16 h) fast; for the *ad libitum* fed group, serum samples were obtained at 2–3 h into the light cycle. Circulating CTRP2 levels were quantified by Western blot. CTRP2 transgenic (Tg) mice and wild-type (WT) control littermates were housed in polycarbonate cages on a 12-h light-dark photocycle with *ad libitum* access to water throughout the study period. Mice were fed *ad libitum* a high-fat diet (HFD; 60% kcal derived from fat, Research Diets, New Brunswick, NJ; D12492) or a matched, control low-fat diet (LFD; 10% kcal derived from fat, Research Diets; D12450B), beginning at 4 weeks of age. HFD was provided for a period of 12 weeks. Blood samples were collected after 12 weeks for serum analysis. For terminal experiments, mice were sacrificed by decapitation under anesthesia. All studies accorded with the recommendations in the Guide for the Care and Use of Laboratory Animals of the National Institutes of Health. All animal experiments were approved by the Animal Care and Use Committee of The Johns Hopkins University School of Medicine (protocol number MO11M49).

### Generation of CTRP2 Transgenic (Tg) Mice

C-terminal HA-epitope tagged CTRP2 was cloned into the *Xho*I site of the pCAGGS vector (25). Expression of CTRP2 transgene was driven by the ubiquitious CAG promoter, which consists of a CMV enhancer element with a chicken β-Actin promoter. The plasmid construct was digested with *Sal*I and *Not*I restriction enzymes (New England Biolabs, Ipswich, MA) and resulting DNA fragments (∼3.3 and 2.5 kb) were separated on a 1% agarose gel. The ∼3.3-kb linear DNA fragment containing the CAG promoter and enhancer, CTRP2-HA transgene, and the rabbit β-globin polyA adenylation signal was excised from the agarose gel, purified, and verified by DNA sequencing. Pronuclear injections were performed; several transgenic founder lines were obtained, and one line was expanded for phenotypic analysis. The transgenic line was generated on a C57BL/6J genetic background. No backcross was necessary. Transgene-negative male littermates were used as WT control mice for transgene-positive male mice throughout the study.

### RT-PCR Analysis

Total RNAs from mouse tissues were isolated with TRIzol® (Invitrogen). One µg of total RNA were reverse-transcribed using Superscript II (Invitrogen). Thirty-cycle PCR was carried out using Hot Start Taq Blue polymerase (Denville); the cycling conditions were as follows: 15 s denaturation at 95°C, 15 s primer annealing at 60°C, and 45 s primer extension at 72°C. Primers used included the following: forward (4F7) 5′- CTGCGGCAGTAGCCGAGC CAAGTCG-3′ and reverse (HA-Tag-R) 5′- TCAAGCGTAGTCTGGGACGTCGTATGGGTA-3′.

### Mouse Serum and Blood Chemistry Analysis

Mouse serum samples were harvested by tail bleeding after overnight fast (∼16 h), or at the indicated time point. Serum samples were separated using microvette® CB 300 (Sarstedt). Glucose concentration was determined at the time of collection with a glucometer (BD bioscience). Serum triglycerides, non-esterified free fatty acids (NEFA), cholesterol, high-density lipoprotein (HDL) cholesterol, and low-density lipoprotein (LDL) cholesterol were measured at the Phenotyping Core at the Johns Hopkins University School of Medicine. Serum insulin, leptin, resistin, adiponectin, PAI-1, and TNF-α (Millipore) were measured according to kit manufacturer’s instructions.

### Body Composition Analysis

Body compositions of WT and Tg mice were determined using a whole-body NMR instrument (EchoMRI, Waco, TX) at the metabolic phenotyping core facility at the Johns Hopkins University School of Medicine. EchoMRI analysis provided values for fat mass, lean mass, and water content.

### Indirect Calorimetry

CTRP2 Tg mice and WT control littermates were used for simultaneous assessments of daily changes in body weight, energy intake (corrected for spillage), and whole-body metabolic profile in an open-flow indirect calorimeter (Oxymax, Columbus Instruments) as described previously [12]. Data were collected for three days to confirm acclimation to the calorimetry chambers (stable body weights and food intakes), and data from the fourth day were analyzed. Rates of oxygen consumption (VO_2_, mL/kg/h) and carbon dioxide production (VCO_2_) were measured for each chamber every 16 min throughout the studies. Respiratory exchange ratio (RER = VCO_2_/VO_2_) was calculated using Oxymax software (v. 4.02) to estimate relative oxidation of carbohydrate (RER = 1.0) versus fat (RER approaching 0.7), not accounting for protein oxidation. Energy expenditure was calculated as EE = VO_2_ x [3.815+(1.232×RER)] [20], and normalized to body mass (kcal/kg/h).

### Intraperitoneal Glucose, Insulin, Pyruvate, and Lipid Tolerance Tests

During glucose tolerance tests (GTT), animals were fasted overnight (∼16 h), and glucose (1 g/kg) was injected intraperitoneally (IP). During insulin tolerance tests (ITT), food was removed 2 h prior to insulin (1.2 U/kg) injection. During pyruvate tolerance tests (PTT), mice were fasted overnight (∼16 h) and then IP-injected with 2 g/kg body weight sodium pyruvate (Sigma-Aldrich) in saline. Blood glucose levels during GTT, ITT, and PTT were measured using a glucometer (BD Pharmingen) at the indicated time points. During lipid tolerance tests (LTT), mice were fasted 12 h and then IP injected with 20% emulsified Intralipid (soybean oil; Sigma; 10 µL/g of body weight). Sera were collected via tail bleed using a Microvette® CB 300 (Sarstedt) at 0, 1, 2, 3, and 5 h post-injection. Serum levels of NEFA and triglycerides were quantified using kits from Wako and Infinity Triglycerides, respectively.

### β3-adrenergic Receptor Agonist Stimulation

Mice were fasted for 2 h, then injected with 1 g/kg body weight of CL316,243 hydrate (β3 adrenergic receptor agonist, Sigma). Blood glucose and sera were harvested via tail bleed at 0, 15, 30, 60, and 120 min post-injection. Serum NEFA levels were measured with a NEFA kit (Wako).

### Isolation of Skeletal Muscle, Liver, and Adipose Tissue

Liver, adipose tissue, and skeletal muscle samples were immediately harvested from euthanized mice and snap-frozen in liquid nitrogen. Homogenized cell lysates were prepared in lysis buffer (20 mM Tris-HCl, 150 mM NaCl, 1 mM EDTA, 0.5% NP-40, and 10% glycerol) containing protease and phosphatase inhibitor cocktails (Sigma-Aldrich). Protein content was quantified using Coomasie Plus protein reagent (Thermo Scientific).

### Immunoblot Analysis

Western blot analysis was carried out as previously described [12]. For tissue lysates, 40 µg from each sample was loaded. For serum samples, one µL-equivalent serum samples were suspended in NuPAGE LDS sample buffer and heated for 5 min at 90°C. Samples were electrophoresed on 10% BisTris NuPAGE gels (Invitrogen, Carlsbad, CA), transferred to 0.2 µm Protran BA83 nitrocellulose membranes (GE Healthcare, Piscataway, NJ), and probed with the CTRP2 primary antibody and appropriate HRP-conjugated secondary antibody. Bands were visualized with Immobilon Western HRP substrate peroxide solution (Millipore, Billerica, MA), captured with MultiImage III FluorChem® Q (Alpha Innotech, San Leandro, CA), and quantified using Alphaview Software (Alpha Innotech).

### Statistical Analysis

All results are expressed as mean ± standard error of the mean (SEM). Statistical analysis was performed with Prism 5 software (GraphPad). Blood chemistry data were analyzed with two-tailed Student’s *t*-tests between CTRP2 Tg and WT control littermates. Average metabolic values (from indirect calorimetry) were calculated within subjects, then averaged across subjects for statistical analysis by Student’s *t*-test. Repeated measures ANOVA were performed on body weights as well as serum glucose, triglyceride, and NEFA measurements in various tolerance tests. Values were considered to be significant at *p*<0.05.

## Results

### Alteration in CTRP2 mRNA and Circulating Levels in Response to Metabolic Perturbation

**Figure 1:**
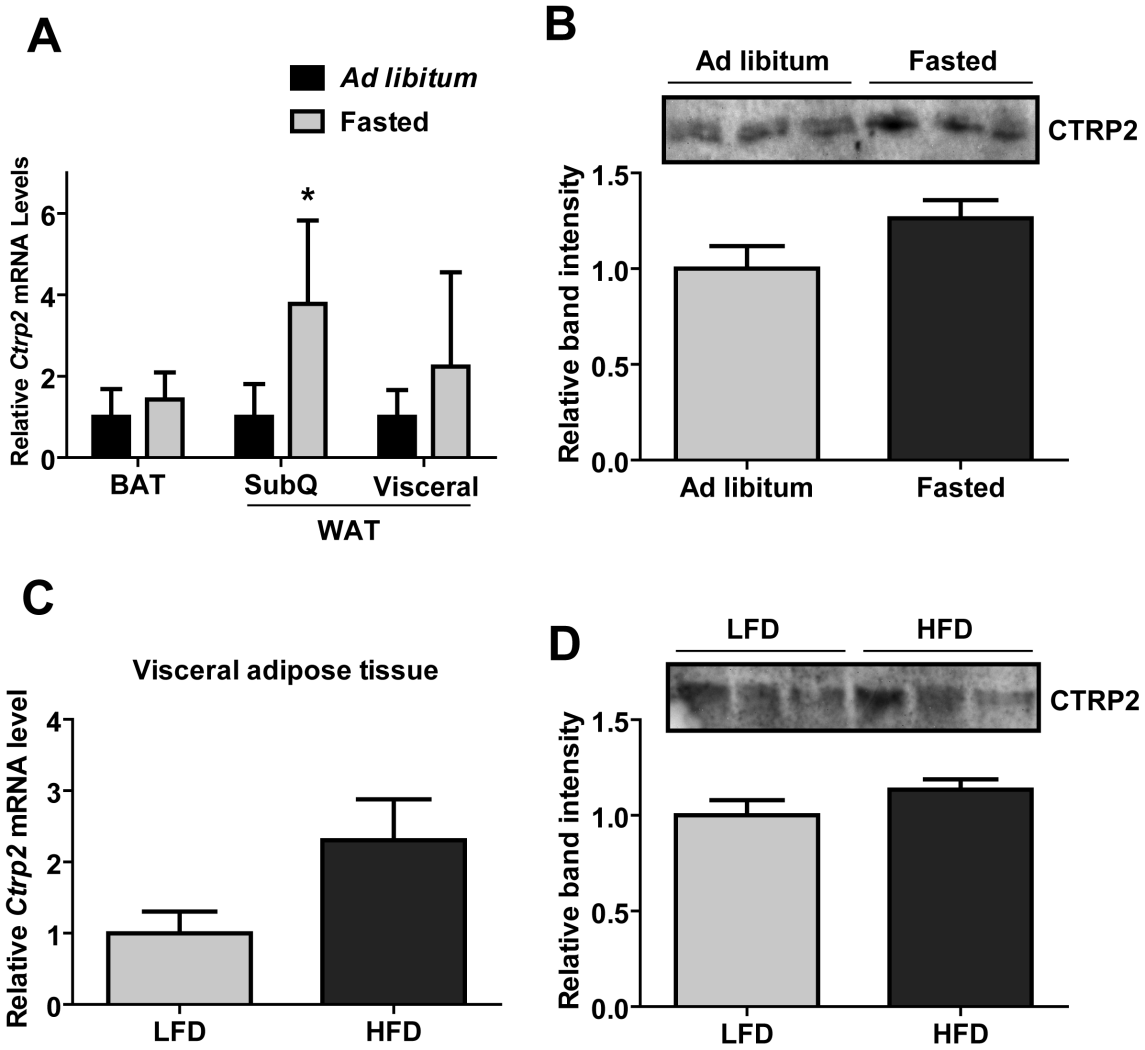
Diet and metabolic state modulate the expression of CTRP2. *A*, Quantitative real-time PCR analyses of *Ctrp2* expression in brown adipose tissue (BAT), visceral (epididymal fat pad), and subcutaneous (SubQ; inguinal fat pad) white adipose tissue from *ad libitum* fed (n = 10) or overnight (16 h) fasted male mice (n = 10). *B*, Quantitative Western blot analysis of CTRP2 serums levels in *ad libitum* fed or overnight (16 h) fasted male mice. Each lane represents data from an independent mouse. *C-D*, Real-time PCR (C) and Western blot (D) analysis of CTRP2 mRNA and serum levels in male C57BL/6 mice fed a high-fat diet (HFD) or a low-fat diet (LFD) for 12 weeks. Each lane on the immunoblot represents data from an independent mouse. Values shown are mean ± SEM. (n = 8–10 mice per group) *p<0.05.

Expression and/or circulating levels of multiple CTRPs change in response to alterations in energy state [5], [6], [7], [10], [12], [13]. We therefore determined whether the expression and plasma levels of CTRP2 change in response to acute and chronic metabolic perturbation. Wild-type C57BL/6 male mice fasted overnight (∼16 h) had ∼4-fold higher transcript levels of *Ctrp2* in the subcutaneous white adipose tissue (subcutaneous fat pad) and 25% higher circulating CTRP2 levels compared to *ad libitum*-fed animals (Fig. 1A,B). In diet-induced obese male mice fed a HFD for 12 weeks, we observed an approximately 2-fold increase in the expression of *Ctrp2* transcript in the visceral white adipose tissue (epididymal fat pad) (Fig. 1C). However, no significant changes were detected in the circulating levels of CTRP2 in HFD-fed mice (Fig. 1D). These results indicate that short-term changes in nutritional state, as well as chronic metabolic perturbation induced by HFD, had an impact on the expression of *Ctrp2*.

### Generation of CTRP2 Transgenic Mice

**Figure 2:**
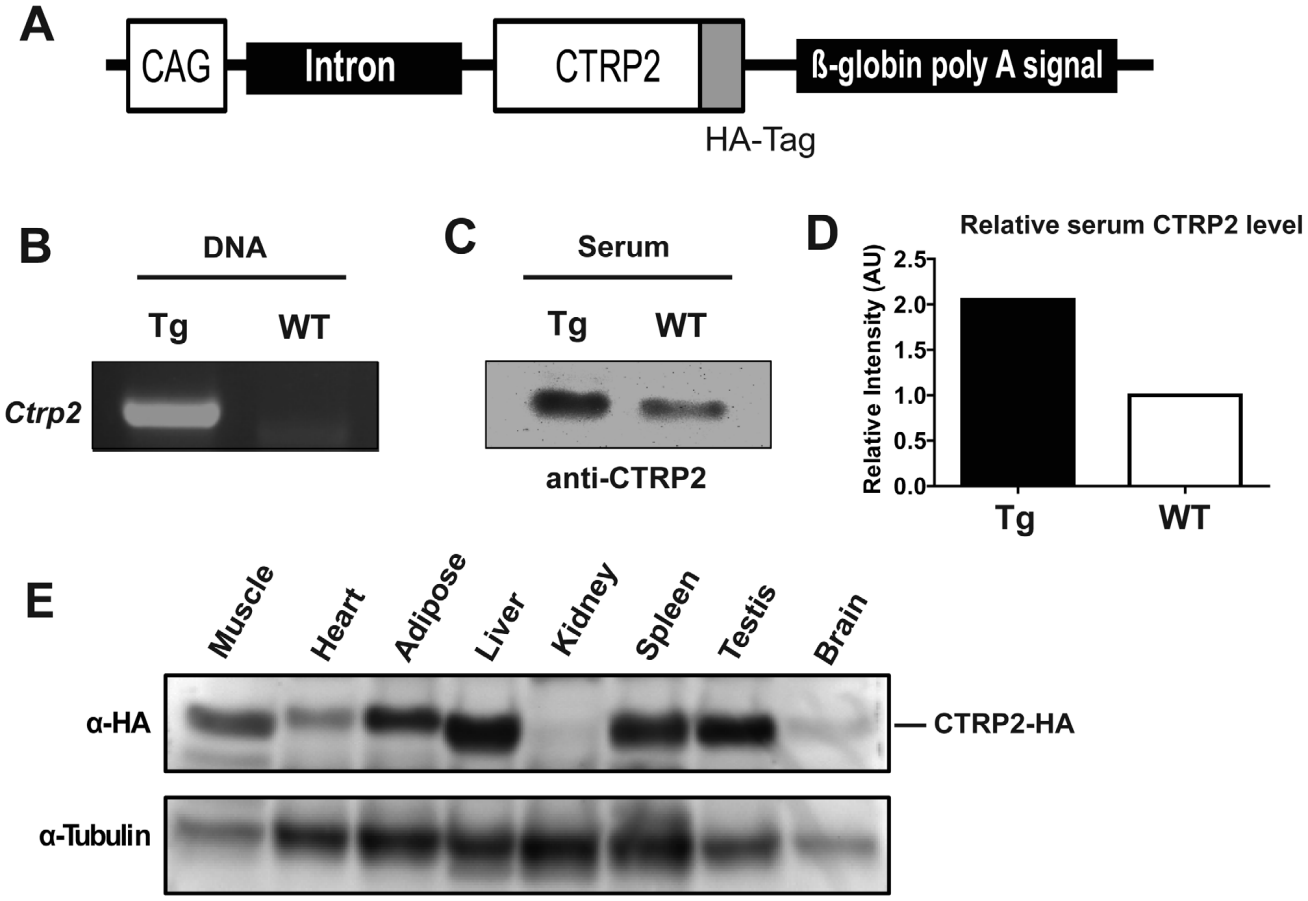
Generation of CTRP2 transgenic mice. *A*, Schematic of *Ctrp*2 transgene construct. HA epitope-tagged *Ctrp2* transgene is driven by the ubiquitous CAG promoter. *B*, RT-PCR analysis demonstrating the presence of *Ctrp2* transgene in Tg mice. *C*, Western blot analysis of CTRP2 in sera from WT and Tg mice. *D*, Quantification of relative band intensity (arbitrary unit) as shown in C. *E*, Western blot analysis of CTRP2-HA protein in mouse tissues.

To identify the *in vivo* metabolic function of CTRP2, we generated a Tg mouse model over-expressing HA epitope-tagged CTRP2. Because CTRP2 is a secreted protein and is normally expressed in multiple tissues and cell types in mice [1], [3], we chose to express the *Ctrp2* transgene using a ubiquitous promoter (Fig. 2A,B). The Tg mouse line had a modest 2-fold higher circulating level of CTRP2 relative to wild-type littermate controls (Fig. 2C,D). At the protein level, we detected the HA-tagged CTRP2 in the skeletal muscle, heart, adipose tissue, liver, spleen, testis, and brain of transgenic animals (Fig. 2E).

### Weight Gain, Food Intake, and Energy Expenditure of CTRP2 Tg Mice Fed a Low- or High-fat Diet

**Figure 3:**
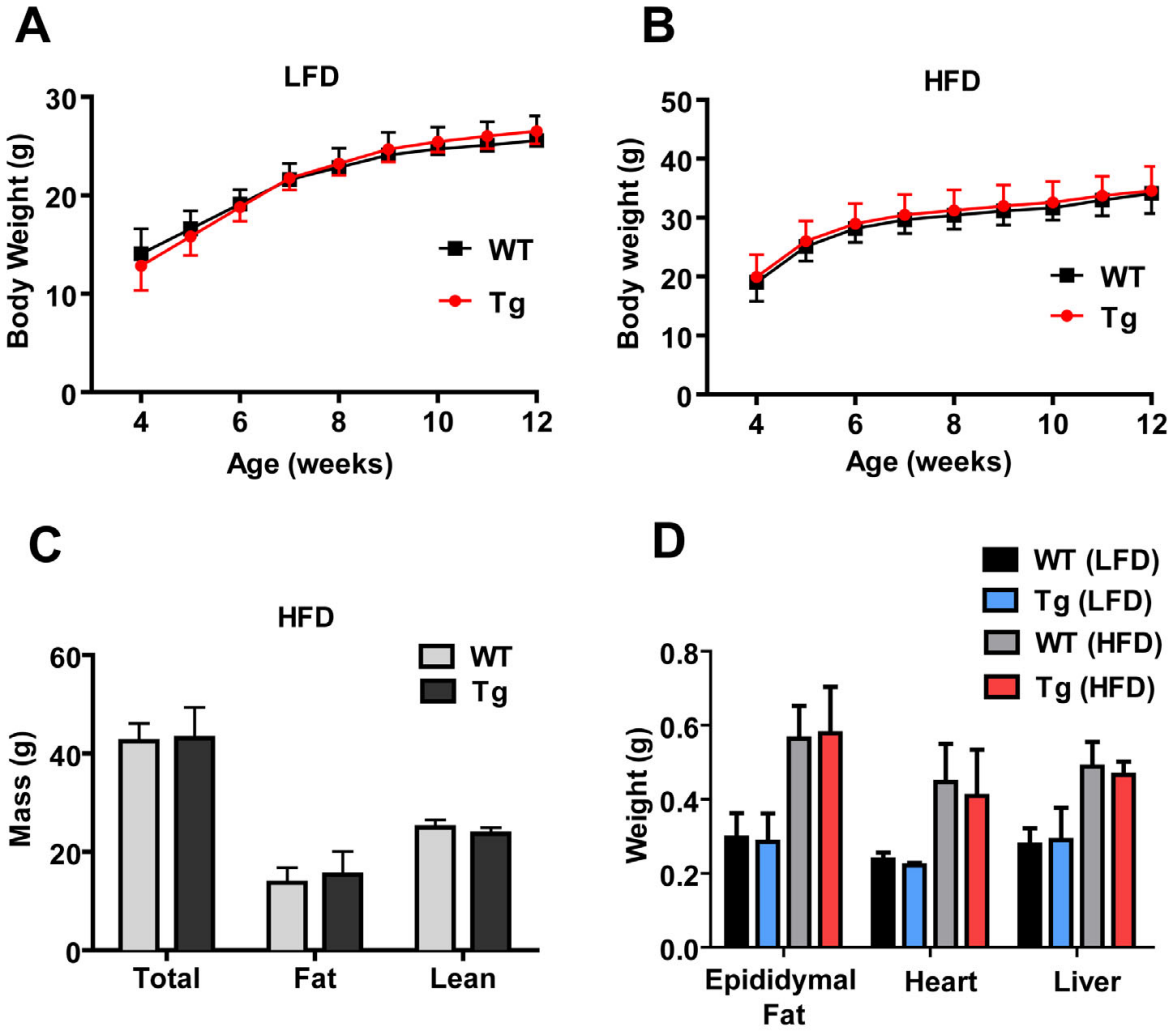
Body weight and body composition analysis of *C*TRP2 transgenic mice. *A*, Body weight gain over time between WT (n = 9) and Tg (n = 11) male mice fed a low-fat diet (LFD). *B*, Body weight gain over time between WT (n = 18) and Tg (n = 24) male mice fed high-fat diet (HFD). *C*, Total, fat, and lean mass in WT (n = 8) and Tg (n = 7) male mice as determined by NMR analysis. *D*, Weight of epipidymal fat pad, heart, and liver of WT (n = 11) and Tg (n = 9) male mice fed an LFD, as well as WT (n = 8) and Tg (n = 10) mice fed an HFD. Values shown are mean ± SEM.

**Figure 4:**
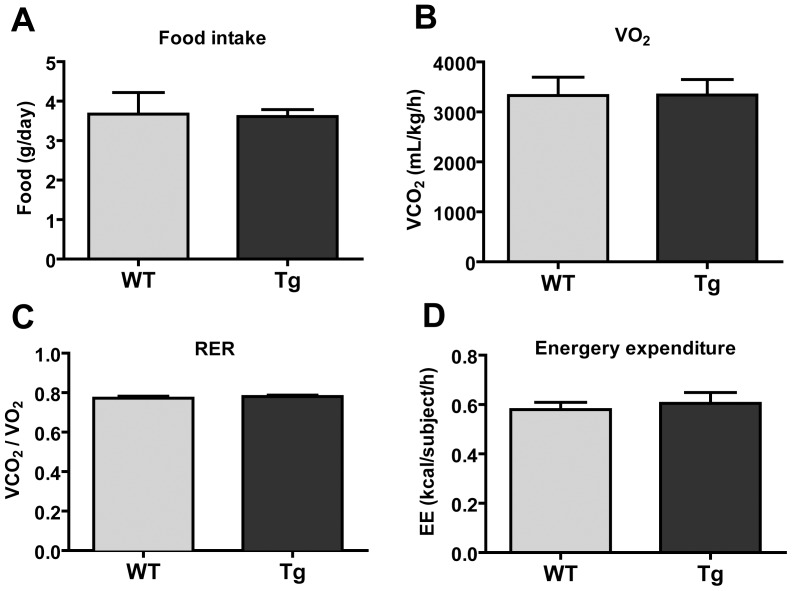
Indirect calorimetry analysis of *C*TRP2 transgenic mice fed a high-fat diet. *A*, Food intake analysis in WT (n = 7) and Tg (n = 8) mice on a high-fat diet. *B-D*, Oxygen consumption (VO_2_; *B*), respiratory exchange ratio (RER = VCO_2_/VO_2_; *C*), and energy expenditure (*D*) of WT (n = 7) and Tg (n = 8) male mice on a high-fat diet as determined by indirect calorimetry. Values shown are mean ± SEM.

CTRP2 Tg mice were born in the expected Mendelian ratio and developed normally with no gross phenotypic abnormality. Body weight gain on LFD (Fig. 3A) and HFD (Fig. 3B) over a period of 8 weeks was indistinguishable between Tg and WT male mice. We obtained similar body weight data on multiple independent cohorts of WT and Tg mice (data not shown). For this reason, we chose to focus our analysis on HFD-fed male mice throughout the study. Consistent with the body weight data, we observed no differences in fat and lean mass between WT and CTRP2 Tg mice (Fig. 3C), nor any differences in heart, liver, and epididymal fat pad weight between the two groups of animals (Fig. 3D). Indirect calorimetry analysis also revealed no differences in food intake, oxygen consumption (a measure of metabolic rate), respiratory exchange ratio (a measure of fat vs. carbohydrate oxidation), or energy expenditure between WT and CTRP2 Tg mice (Fig. 4).

### Improved Insulin Tolerance in Obese CTRP2 Tg Mice

**Figure 5:**
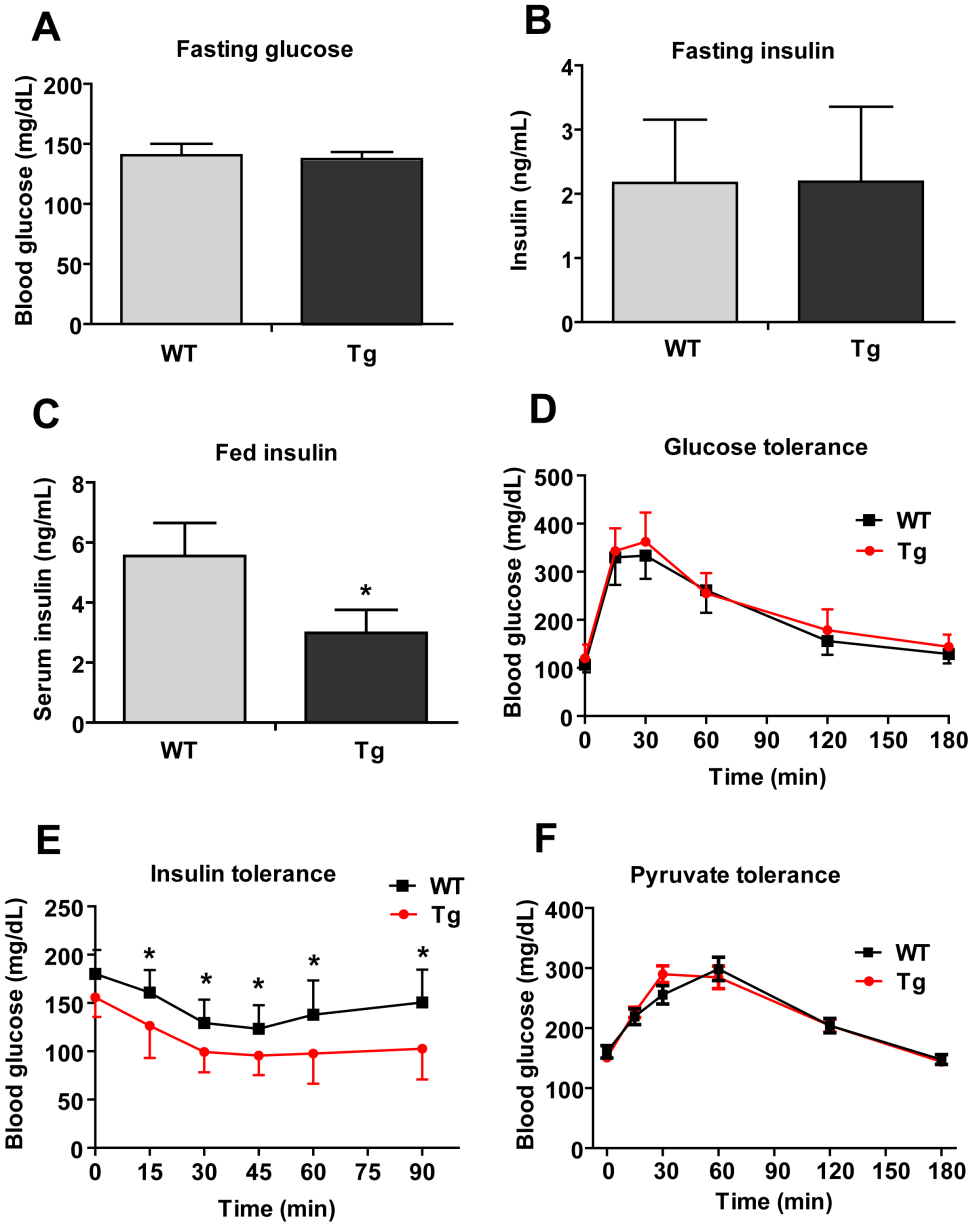
Insulin sensitivity in CTRP2 transgenic mice. *A–B*, Fasting glucose (*A*) and insulin (*B*) levels in WT (n = 8) and Tg (n = 11) mice. *C*, Serum insulin levels in the fed state in WT (n = 13) and Tg (n = 8) mice. *D-E*, Glucose (*D*) and insulin (*E*) tolerance test in WT (n = 9) and Tg (n = 10) mice. *F*, Pyruvate tolerance test in WT (n = 10) and Tg (n = 8) mice. Values shown are mean ± SEM. *p<0.05 vs. WT.

We observed no differences in fasting glucose and insulin levels between WT and CTRP2 Tg mice (Fig. 5A,B). However, we consistently noted that CTRP2 Tg mice had lower blood glucose in the fed state (Fig. 5C). This was confirmed in separate cohorts of WT and Tg mice, suggesting that the Tg animals may be more insulin-sensitive in the fed state. We performed glucose tolerance tests in overnight-fasted mice and observed no difference in the rate of glucose disposal in the peripheral tissues between WT and CTRP2 Tg animals (Fig. 5D). In contrast, during insulin tolerance tests the rate of glucose disposal in the peripheral tissues was greater in CTRP2 Tg mice relative to WT littermate controls (Fig. 5E), consistent with improved insulin action. Improved insulin tolerance was seen only in the HFD-fed Tg mice and not the LFD-fed Tg mice (data not shown). To assess possible changes in hepatic insulin sensitivity, we performed a pyruvate tolerance test. In overnight-fasted mice where hepatic glycogen has been depleted, pyruvate (a gluconeogenic substrate) will be converted to glucose in the liver to buffer blood glucose [21]. Because insulin suppresses hepatic gluconeogenesis, the rate of hepatic glucose output in response to pyruvate administration indicates the extent of insulin sensitivity in the liver. Here, when overnight-fasted mice were given sodium pyruvate, no differences in hepatic glucose production were observed between WT and CTRP2 Tg mice (Fig. 5F).

### Improved Lipid Tolerance in Obese CTRP2 Tg Mice

**Figure 6:**
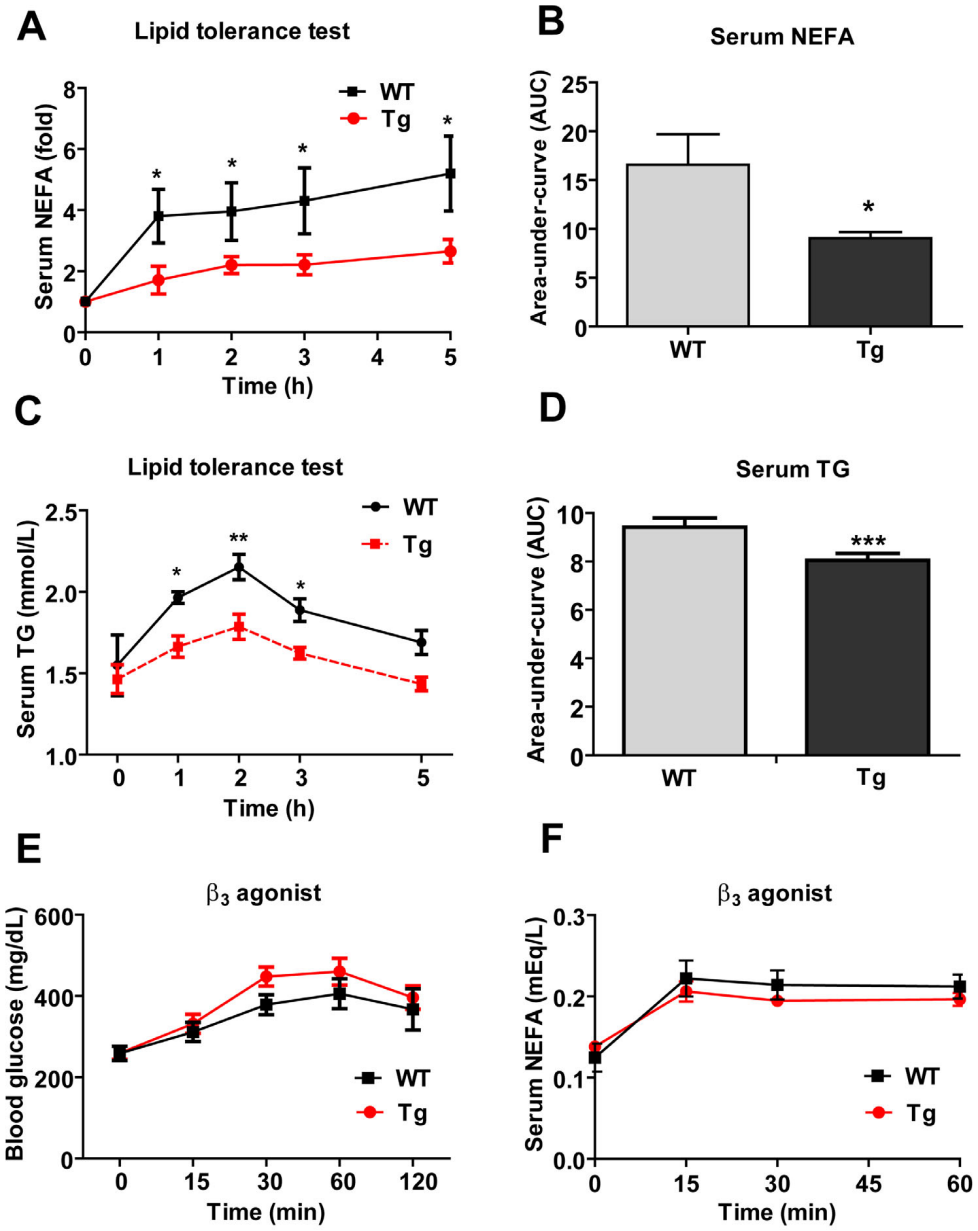
Lipid tolerance in CTRP2 transgenic mice. *A–B*, Serum non-esterified free fatty acid (NEFA) levels in WT (n = 9) and Tg (n = 6) mice over time in response to emulsified lipid infusion. *C–D*, Serum triglyceride (TG) levels in WT (n = 9) and Tg (n = 6) mice over time in response to emulsified lipid infusion. *E-F*, Blood glucose and serum NEFA over time in WT (n = 8) and Tg (n = 11) in response to β3 adrenergic receptor agonist administration. Values shown are mean ± SEM. *p<0.05 vs. WT. **p<0.01.

To determine whether the HFD-fed WT and Tg mice differ in their capacity to handle acute lipid challenge we performed a lipid tolerance test. Fasted mice were IP-injected with emulsified lipids, mimicking the sudden rise of plasma lipids in response to food intake [22], and the circulating levels of NEFA and triglycerides were quantified over time. The CTRP2 Tg mice demonstrated a significantly greater capacity to clear an acute rise in free fatty acids and triglycerides in response to emulsified lipid infusion compared to WT controls (Fig. 6A–D), suggesting an enhanced capacity to handle lipid load. Improvements in lipid tolerance were only seen in the HFD-fed and not in the LFD-fed Tg mice (data not shown). Next, we determined whether CTRP2 Tg mice had an altered capacity to mobilize free fatty acids from adipose tissue in response to fasting or sympathetic input (via the β3-adrenergic receptor). Overnight fast did not lead to any differences in serum non-esterified free fatty acids (NEFA) (Table 1). Administration of a β3-adrenergic receptor agonist (CL316,243) to WT and Tg animals also revealed no differences in agonist-induced rise in blood glucose (Fig. 6E) or the release of free fatty acids from adipose tissue triglycerides (Fig. 6F).

**Table 1:** Serum lipid and adipokine profiles between WT and CTRP2 Tg mice.

	WT	Tg
NEFA (mEq/L)	0.82±0.08	0.76±0.06
Triglycerides (mg/dL)	58.6±3.9	59.1±3.1
Total cholesterol (mg/dL)	99.1±8.4	106.4±9.9
HDL-cholesterol (mg/dL)	44.6±3.1	51.2±4.7
LDL-cholesterol (mg/dL)	42.8±6.2	43.4±5.5
Leptin (ng/mL)	7.2±1.6	6.3±1.3
Adiponectin (µg/mL)	14.6±1.0	12.5±1.7
Resistin (ng/mL)	1.4±0.15	1.37±0.10
TNF-α (pg/mL)	6.0±0.2	6.2±0.2
PAI-1 (ng/mL)	2.78±0.6	2.69±0.5

Quantification of fasting serum lipid metabolites and adipokines levels in WT (n = 10) and Tg (n = 10) male mice.

### Serum Lipid and Adipokine Profiles of Obese CTRP2 Tg Mice

Circulating levels of lipid metabolites and adipokines are tightly linked to metabolic state. We performed blood chemistry analysis on the HFD-fed WT and CTRP2 Tg mice and observed no differences in serum lipid (NEFA, triglycerides, total cholesterol, HDL-cholesterol, and LDL-cholesterol) or adipokine (leptin, adiponectin, resistin, TNF-α, and PAI-1) levels between the two groups of animals (Table 1). Similarly, we observed no differences in serum metabolite and adipokine profiles in the LFD-fed WT and Tg mice (data not shown).

## Discussion

Using a transgenic mouse model, we sought to determine a role for CTRP2 in regulating whole-body metabolism *in vivo*. Whether in the context of an LFD or the metabolic challenge of an HFD, CTRP2 over-expression in Tg mice had little impact on the multiple metabolic parameters examined. These included food intake, body weight, energy expenditure, and serum lipid and adipokine profiles. However, insulin tolerance tests suggested that CTRP2 Tg mice do exhibit improved insulin action, indicated by an increased rate of glucose disposal in the peripheral tissues in response to insulin administration. The improvements in insulin tolerance relative to WT littermate controls were only seen in the CTRP2 Tg mice fed an HFD and not if they were fed the control LFD. Similarly, the HFD-fed CTRP2 Tg mice showed an enhancement in the capacity of Tg animals to handle an acute lipid challenge, as indicated by a greater rate of free fatty acids and triglyceride clearance in response to emulsified lipid infusion.

Despite improvements in acute lipid tolerance, CTRP2 Tg mice chronically fed an HFD did not show any differences in body weight or fat mass compared to WT littermate controls. Our previous *in vitro* studies suggest that recombinant CTRP2 activates AMPK signaling to enhance fat oxidation in mouse C2C12 myotubes [1]. Overexpressing CTRP2 in mice, however, did not result in higher basal AMPK activation. We did not observe any differences in the phosphorylation and activation of AMPKα, nor its downstream target, acetyl-CoA carboxylase (ACC) in the skeletal muscle, liver, or adipose tissue of WT and Tg animals (Fig. S1). Nor did we observe any differences in fat oxidation in CTRP2 Tg mice, as indicated by the VO_2_ and respiratory exchange ratio (RER). While *in vitro* studies using cultured cells and purified recombinant protein are informative, our Tg mouse study highlights the complexity of the *in vivo* milieu, where cells receive multiple positive and negative inputs concurrently, a physiological context that is difficult to capture *in vitro*. Interestingly, we did observe a 2-fold higher basal level of p44/42-MAPK activation, as judged by Thr-202/Tyr-204 phosphorylation, in the liver of CTRP2 Tg mice relative to WT controls (data not shown); the significance of this observation is unclear since the pyruvate tolerance test we performed to evaluate hepatic insulin sensitivity was not different between WT and Tg mice. Given that energy balance is governed by multiple homeostatic mechanisms, the caveats of over-expressing CTRP2 in mice include potential physiologic compensations that could mask the true impact of CTRP2 on systemic glucose and lipid metabolism. Alternatively, there may be a threshold effect for CTRP2; the presence of more CTRP2 protein due to transgenic overexpression would not lead to further improvements in metabolic profile of these animals. Employing a CTRP2 loss-of-function mouse model in future studies will help clarify and establish its physiologic role in modulating energy metabolism.

In contrast to the modest phenotypes seen in CTRP2 Tg mice, overexpressing the related CTRP family members–CTRP1, CTRP3, and CTRP9–in mice using a similar transgenic approach has resulted in significant improvements in the metabolic profiles of these animals compared to WT littermate controls [10], [11], [12]. When fed an HFD, CTRP1 Tg mice have lower body weights due to increased energy expenditure, leading to greater systemic insulin sensitivity [10]. CTRP1 Tg mice also have higher basal AMPK activation in the skeletal muscle, leading to enhanced fat oxidation [10]. While no differences in body weight, energy expenditure, and glucose tolerance are observed between CTRP3 Tg and WT mice, the transgenic animals are strikingly resistant to the development of fatty liver (steatosis) in response to high-fat feeding [11]. Overexpressing CTRP3 in mice suppresses the expression of genes (*Gpat*, *Agpat*, *Dgat*) involved in triglyceride synthesis, leading to decreased triglyceride accumulation in the liver of the Tg animals [11]. Of the CTRP Tg mouse models described to date, the most dramatic and striking phenotypes are observed in mice overexpressing CTRP9 [12]. CTRP9 Tg mice are lean and resistant to weight gain when fed an HFD. All the metabolic dysfunctions (insulin resistance, obesity, fatty liver, dyslipidemia) associated with high-fat feeding are prevented in CTRP9 Tg mice [12]. These remarkable metabolic phenotypes are due to a combination of reduced caloric intake and increased energy metabolism [12]. The present study and recent findings using transgenic mouse models suggest that each CTRP has a unique role in regulating glucose and/or lipid metabolism *in vivo*, consistent with their high degree of conservation throughout vertebrate evolution [9].

## Supporting Information

Figure S1 Basal AMPKα (Thr-172) and ACC (ser-79) phosphorylations in the skeletal muscle, liver, and adipose tissue of WT and CTRP2 Tg mice fed a high-fat diet (n = 3 per group). Each lane represents tissue sample from a different mouse. Replicate blots were probed for phospho and total AMPK and ACC. P-ACC, phospho Acetyl-CoA carboxylase; p-AMPK, phospho AMP-activated protein kinase.
(TIF)

## References

[1] Wong GW, Wang J, Hug C, Tsao TS, Lodish HF (2004) A family of Acrp30/adiponectin structural and functional paralogs. Proc Natl Acad Sci U S A 101: 10302–10307. 15231994 10.1073/pnas.0403760101 PMC478567

[2] Wong GW, Krawczyk SA, Kitidis-Mitrokostas C, Ge G, Spooner E, et al (2009) Identification and characterization of CTRP9, a novel secreted glycoprotein, from adipose tissue that reduces serum glucose in mice and forms heterotrimers with adiponectin. FASEB J 23: 241–258. 18787108 10.1096/fj.08-114991 PMC2626616

[3] Wong GW, Krawczyk SA, Kitidis-Mitrokostas C, Revett T, Gimeno R, et al (2008) Molecular, biochemical and functional characterizations of C1q/TNF family members: adipose-tissue-selective expression patterns, regulation by PPAR-gamma agonist, cysteine-mediated oligomerizations, combinatorial associations and metabolic functions. Biochem J 416: 161–177. 18783346 10.1042/BJ20081240 PMC3936483

[4] Wei Z, Peterson JM, Lei X, Cebotaru L, Wolfgang MJ, et al (2012) C1q/TNF-related protein-12 (CTRP12), a novel adipokine that improves insulin sensitivity and glycemic control in mouse models of obesity and diabetes. J Biol Chem 287: 10301–10315. 22275362 10.1074/jbc.M111.303651 PMC3322967

[5] Wei Z, Peterson JM, Wong GW (2011) Metabolic regulation by C1q/TNF-related protein-13 (CTRP13): activation OF AMP-activated protein kinase and suppression of fatty acid-induced JNK signaling. J Biol Chem 286: 15652–15665. 21378161 10.1074/jbc.M110.201087 PMC3091174

[6] Wei Z, Seldin MM, Natarajan N, Djemal DC, Peterson JM, et al (2013) C1q/Tumor Necrosis Factor-related Protein 11 (CTRP11), a Novel Adipose Stroma-derived Regulator of Adipogenesis. J Biol Chem 288: 10214–10229. 23449976 10.1074/jbc.M113.458711 PMC3624406

[7] Seldin MM, Peterson JM, Byerly MS, Wei Z, Wong GW (2012) Myonectin (CTRP15), a novel myokine that links skeletal muscle to systemic lipid homeostasis. J Biol Chem 287: 11968–11980. 22351773 10.1074/jbc.M111.336834 PMC3320944

[8] Byerly MS, Petersen PS, Ramamurthy S, Seldin MM, Lei X, et al.. (2013) C1q/TNF-related protein 4 (CTRP4) is a unique secreted protein with two tandem C1q domains that functions in the hypothalamus to modulate food intake and body weight. J Biol Chem In press. 10.1074/jbc.M113.506956 PMC3924272 24366864

[9] Seldin MM, Tan SY, Wong GW (2013) Metabolic function of the CTRP family of hormones. Rev Endocr Metab Disord in press. 10.1007/s11154-013-9255-7 PMC3931758 23963681

[10] Peterson JM, Aja S, Wei Z, Wong GW (2012) C1q/TNF-related protein-1 (CTRP1) enhances fatty acid oxidation via AMPK activation and ACC inhibition. J Biol Chem 287: 1576–1587. 22086915 10.1074/jbc.M111.278333 PMC3256898

[11] Peterson JM, Seldin MM, Wei Z, Aja S, Wong GW (2013) CTRP3 attenuates diet-induced hepatic steatosis by regulating triglyceride metabolism. Am J Physiol Gastrointest Liver Physiol 305: G214–224. 23744740 10.1152/ajpgi.00102.2013 PMC3742855

[12] Peterson JM, Wei Z, Seldin MM, Byerly MS, Aja S, et al (2013) CTRP9 transgenic mice are protected from diet-induced obesity and metabolic dysfunction. Am J Physiol Regul Integr Comp Physiol 305: R522–533. 23842676 10.1152/ajpregu.00110.2013 PMC3763026

[13] Peterson JM, Wei Z, Wong GW (2010) C1q/TNF-related Protein-3 (CTRP3), a Novel Adipokine That Regulates Hepatic Glucose Output. J Biol Chem 285: 39691–39701. 20952387 10.1074/jbc.M110.180695 PMC3000950

[14] Enomoto T, Ohashi K, Shibata R, Higuchi A, Maruyama S, et al (2011) Adipolin/C1qdc2/CTRP12 functions as an adipokine that improves glucose metabolism. J Biol Chem 286: 34552–34558. 21849507 10.1074/jbc.M111.277319 PMC3186379

[15] Byerly MS, Swanson R, Wei Z, Seldin MM, McCulloh PS, et al (2013) A Central Role for C1q/TNF-Related Protein 13 (CTRP13) in Modulating Food Intake and Body Weight. PLoS One 8: e62862. 23638159 10.1371/journal.pone.0062862 PMC3636217

[16] Su H, Yuan Y, Wang XM, Lau WB, Wang Y, et al (2013) Inhibition of CTRP9, a novel and cardiac-abundantly expressed cell survival molecule, by TNFalpha-initiated oxidative signaling contributes to exacerbated cardiac injury in diabetic mice. Basic Res Cardiol 108: 315–326. 23212557 10.1007/s00395-012-0315-z PMC6408949

[17] Uemura Y, Shibata R, Ohashi K, Enomoto T, Kambara T, et al (2013) Adipose-derived factor CTRP9 attenuates vascular smooth muscle cell proliferation and neointimal formation. FASEB J 27: 25–33. 22972916 10.1096/fj.12-213744

[18] Kambara T, Ohashi K, Shibata R, Ogura Y, Maruyama S, et al (2012) CTRP9 protein protects against myocardial injury following ischemia-reperfusion through AMP-activated protein kinase (AMPK)-dependent mechanism. J Biol Chem 287: 18965–18973. 22514273 10.1074/jbc.M112.357939 PMC3365930

[19] Zheng Q, Yuan Y, Yi W, Lau WB, Wang Y, et al (2011) C1q/TNF-Related Proteins, A Family of Novel Adipokines, Induce Vascular Relaxation Through the Adiponectin Receptor-1/AMPK/eNOS/Nitric Oxide Signaling Pathway. Arterioscler Thromb Vasc Biol 31: 2616–2623. 21836066 10.1161/ATVBAHA.111.231050 PMC3197867

[20] Lusk G (1928) The elements of the science of nutrition. Philadelphia London: W. B. Saunders company.

[21] Cherrington AD (1999) Banting Lecture 1997. Control of glucose uptake and release by the liver in vivo. Diabetes 48: 1198–1214. 10331429 10.2337/diabetes.48.5.1198

[22] Kim JY, van de Wall E, Laplante M, Azzara A, Trujillo ME, et al (2007) Obesity-associated improvements in metabolic profile through expansion of adipose tissue. J Clin Invest 117: 2621–2637. 17717599 10.1172/JCI31021 PMC1950456

