# Supplementary figures and images for: CTRP2 Overexpression Improves Insulin and Lipid Tolerance in Diet-Induced Obese Mice
Source: PLoS One. 2014 Feb 20;9(2):e88535. doi: 10.1371/journal.pone.0088535 (PMC3930646; doi:10.1371/journal.pone.0088535)

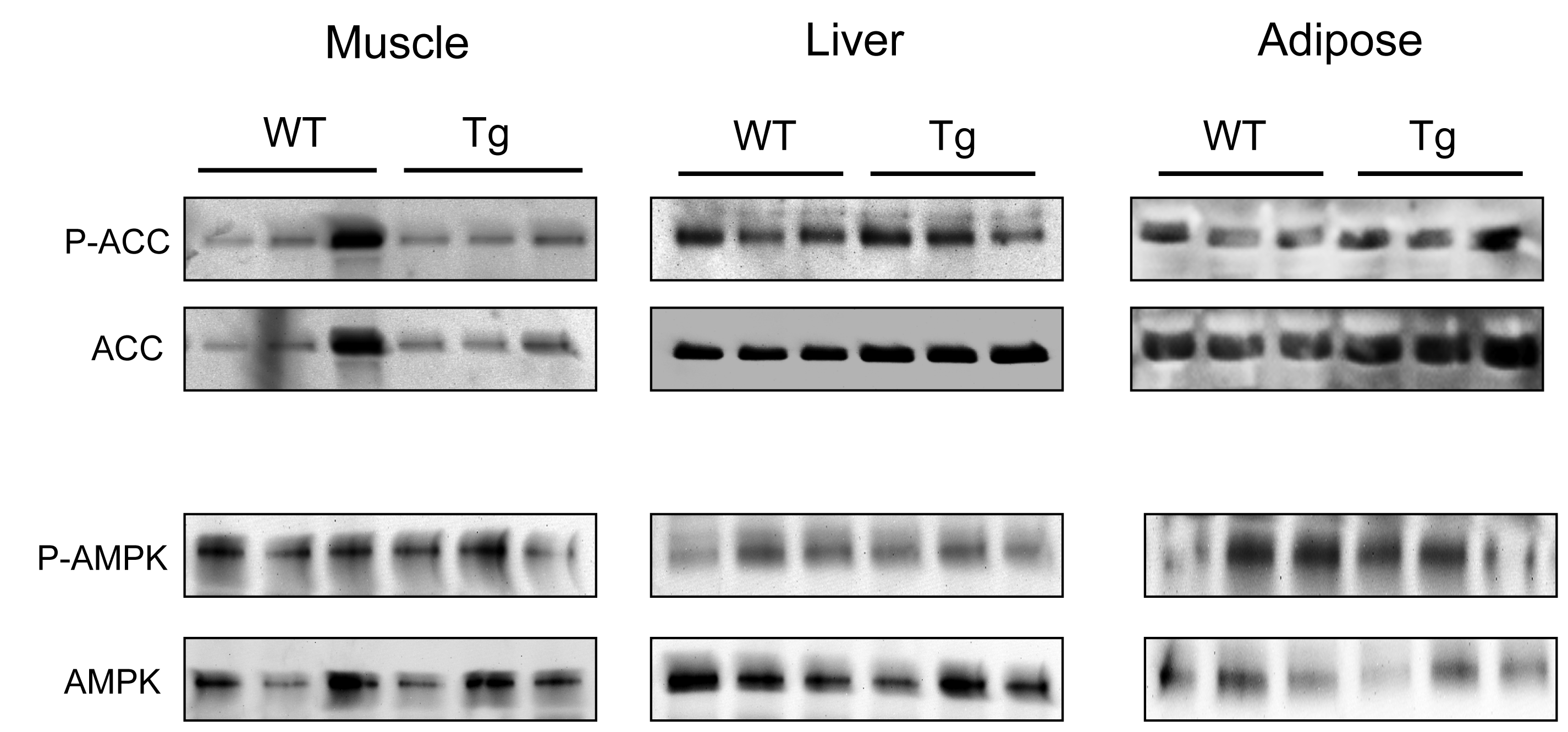

Supplement: Figure S1 — Basal AMPKα (Thr-172) and ACC (ser-79) phosphorylations in the skeletal muscle, liver, and adipose tissue of WT and CTRP2 Tg mice fed a high-fat diet (n = 3 per group). Each lane represents tissue sample from a different mouse. Replicate blots were probed for phospho and total AMPK and ACC. P-ACC, phospho Acetyl-CoA carboxylase; p-AMPK, phospho AMP-activated protein kinase. (TIF) [file pone.0088535.s001.tif]
